# Revolutionizing early Alzheimer's disease and mild cognitive impairment diagnosis: a deep learning MRI meta-analysis

**DOI:** 10.1055/s-0044-1788657

**Published:** 2024-08-15

**Authors:** Li-xue Wang, Yi-zhe Wang, Chen-guang Han, Lei Zhao, Li He, Jie Li

**Affiliations:** 1 Beijing Tsinghua Changgung Hospital, Department of Radiology, Beijing, China.; 2 Tsinghua University, School of Clinical Medicine, Beijing, China.; 3 Beijing Tsinghua Changgung Hospital, Department of Information Administration, Beijing, China.

**Keywords:** Alzheimer Disease, Cognitive Dysfunction, Magnetic Resonance Imaging, Deep Learning, Meta-Analysis, Doença de Alzheimer, Disfunção Cognitiva, Imageamento por Ressonância Magnética, Aprendizado Profundo, Metanálise

## Abstract

**Background** The early diagnosis of Alzheimer's disease (AD) and mild cognitive impairment (MCI) remains a significant challenge in neurology, with conventional methods often limited by subjectivity and variability in interpretation. Integrating deep learning with artificial intelligence (AI) in magnetic resonance imaging (MRI) analysis emerges as a transformative approach, offering the potential for unbiased, highly accurate diagnostic insights.

**Objective** A meta-analysis was designed to analyze the diagnostic accuracy of deep learning of MRI images on AD and MCI models.

**Methods** A meta-analysis was performed across PubMed, Embase, and Cochrane library databases following the Preferred Reporting Items for Systematic Reviews and Meta-Analyses (PRISMA) guidelines, focusing on the diagnostic accuracy of deep learning. Subsequently, methodological quality was assessed using the QUADAS-2 checklist. Diagnostic measures, including sensitivity, specificity, likelihood ratios, diagnostic odds ratio, and area under the receiver operating characteristic curve (AUROC) were analyzed, alongside subgroup analyses for T1-weighted and non-T1-weighted MRI.

**Results** A total of 18 eligible studies were identified. The Spearman correlation coefficient was -0.6506. Meta-analysis showed that the combined sensitivity and specificity, positive likelihood ratio, negative likelihood ratio, and diagnostic odds ratio were 0.84, 0.86, 6.0, 0.19, and 32, respectively. The AUROC was 0.92. The quiescent point of hierarchical summary of receiver operating characteristic (HSROC) was 3.463. Notably, the images of 12 studies were acquired by T1-weighted MRI alone, and those of the other 6 were gathered by non-T1-weighted MRI alone.

**Conclusion** Overall, deep learning of MRI for the diagnosis of AD and MCI showed good sensitivity and specificity and contributed to improving diagnostic accuracy.

## Resumo

**Antecedentes** O diagnóstico precoce da doença de Alzheimer (DA) e do comprometimento cognitivo leve (CCL) continua sendo um desafio significativo na neurologia, com métodos convencionais frequentemente limitados pela subjetividade e variabilidade na interpretação. A integração da aprendizagem profunda com a inteligência artificial (IA) na análise de imagens de ressonância magnética surge como uma abordagem transformadora, oferecendo o potencial para *insights* diagnósticos imparciais e altamente precisos.

**Objetivo** Uma metanálise foi projetada para analisar a precisão diagnóstica do aprendizado profundo de imagens de ressonância magnética em modelos de DA e CCL.

**Métodos** Uma metanálise foi realizada nos bancos de dados das bibliotecas PubMed, Embase e Cochrane seguindo as diretrizes *Preferred Reporting Items for Systematic Reviews and Meta-Analyses* (PRISMA), com foco na precisão diagnóstica do aprendizado profundo. Posteriormente, a qualidade metodológica foi avaliada por meio do *checklist* QUADAS-2. Medidas diagnósticas, incluindo sensibilidade, especificidade, razões de verossimilhança, razão de chances diagnósticas e área sob a curva característica de operação do receptor ( *area under the receiver operating characteristic curve* [AUROC]) foram analisadas, juntamente com análises de subgrupo para ressonância magnética ponderada em T1 e não ponderada em T1.

**Resultados** Um total de 18 estudos elegíveis foram identificados. O coeficiente de correlação de Spearman foi de -0,6506. A metanálise mostrou que a sensibilidade e a especificidade combinadas, a razão de verossimilhança positiva, a razão de verossimilhança negativa e a razão de chances de diagnóstico foram 0,84, 0,86, 6,0, 0,19 e 32, respectivamente. A AUROC foi de 0,92. O ponto quiescente do resumo hierárquico da característica de operação do receptor ( *hierarchical summary of receiver operating characteristic* [HSROC]) foi 3,463. Notavelmente, as imagens de 12 estudos foram adquiridas apenas por ressonância magnética ponderada em T1, e as dos outros 6 foram obtidas apenas por ressonância magnética não ponderada em T1.

**Conclusão** Em geral, a aprendizagem profunda da ressonância magnética para o diagnóstico de DA e CCL mostrou boa sensibilidade e especificidade e contribuiu para melhorar a precisão diagnóstica.

## INTRODUCTION

A study has shown that Alzheimer's disease (AD), the most common neurodegenerative cause in patients with dementia, is primarily characterized by a decline in brain function in multiple areas, including memory, reasoning, and language. 1 Patients with AD account for 50–70% of all patients with neurodegenerative dementia. Unfortunately, with the trend towards an aging population, the number of patients with AD is expected to surge. Xia, P. et al. investigated that there were approximately 6.08 million cases of AD in the United States in 2017, but the number is expected to reach 1.5 billion by 2060. 2 Besides, according to Klyucherev, T. O. et al., the healthcare expenditure on caring for dementia patients was estimated to be $700 million in the United States in 2020, and the economic cost spent on AD patients exceeded the cost of cancer or cardiovascular disease, 3 which brought great trouble to mankind. Based on previous studies, it is known that AD is a complex, heterogeneous, and progressive disease. The predominant molecular mechanism of AD is the formation of toxic amyloid-β oligomers and protein aggregates, as well as the formation of neurofibrillary tangles composed of Tau Protein Hyperphosphorylation, thereby leading to region-specific reduction of brain glucose metabolism synaptic dysfunction, and mitochondrial dysfunction. 4 There are 4 main stages in the development of AD, including the presymptomatic stage, the prodromal stage of mild cognitive impairment (MCI), and the clinical form of AD. Although the efficacy of pharmacological treatments for AD is unsatisfactory, cognitive and physical activity treatments in the early stages of AD, such as the MCI period, play a positive role in reducing cognitive decline. 5 Therefore, researchers have concluded that early detection of brain changes associated with AD is the key to more effective clinical interventions and prevention of disease progression and morbidity. 6 Hence, early diagnosis is particularly important for AD patients.

Currently, imaging modalities are used to identify the early diagnostic and prognostic factors of AD in clinical practice. Among them, magnetic resonance imaging (MRI) is an essential method often used to explore the neuropathological mechanisms and clinical diagnosis of AD and MCI. 7 MRI, as morphometry primarily based on voxel, is a valid and noninvasive method to quantify volumetric brain atrophy caused by severe neuronal loss. Moreover, the integrity of white matter fiber bundles within axonal projections can be assessed *in vivo* using diffusion tensor imaging. 8 MRI can clearly define the pattern of brain injury. On the one hand, MRI measurements of medial temporal lobe atrophy are considered to be a valid marker for clinical AD diagnosis, which can distinguish AD from other brain disorders. On the other hand, MRI can also determine the risk of developing AD or other brain abnormalities from MCI. 9 Due to the high utility of MRI for the diagnosis of AD and MCI, several rating scales have also been established to aid in diagnosis. 10 Besides, a number of studies have begun to develop computer software for automatic MRI assessment to increase diagnostic accuracy and consistency. However, in the process of clinical transformation, the reliability of automatic diagnostic results is not ideal as a wide range of disease characteristics are not specific. Luckily, with the development of artificial intelligence (AI) technology and the popularity of medical applications, diagnostic algorithms can be built through deep learning of clinical data. Such algorithms can be optimized repeatedly to minimize errors.

Moreover, there are many studies today that deep learning—a branch of AI that utilizes layered neural networks to model and analyze vast amounts of data—based on MRI images is employed for the diagnosis of AD. This method is able to reliably capture and quantify a variety of subtle MRI changes throughout the brain, and then amplify the complexity and heterogeneity of AD and brain aging. 11 In addressing the complexities of AD and MCI diagnosis, the role of deep learning cannot be overstated. 12 Specifically, within the domain of MRI analysis, deep learning techniques have the potential to revolutionize diagnostic processes by autonomously identifying and extracting pivotal features from imaging data, a task that traditionally required extensive manual intervention. 13 The application of these advanced AI models to neuroimaging data has been shown to significantly enhance the detection of early and subtle neuroanatomical changes indicative of AD and MCI, thereby offering promising avenues for timely and accurate diagnoses. 14 Nonetheless, no studies have evaluated the accuracy of deep learning in MRI for diagnosing AD and MCI. The objective of this paper was to collect relevant studies on deep learning of MRI for diagnosis of AD and MCI and conduct a meta-analysis to verify the diagnostic accuracy of these articles. These findings have the potential to significantly enhance the diagnostic process for Alzheimer's disease and mild cognitive impairment, leading to earlier and more accurate identification of these conditions. Such advancements could greatly improve patient outcomes by enabling timely intervention and personalized treatment strategies, ultimately contributing to a better quality of life and slower disease progression.

## METHODS

### Literature search strategy

This study was conducted on the grounds of the regulations in the Preferred Reporting Items for Systematic Reviews and Meta-Analyses (PRISMA). 15 Taking MRI, deep learning, AD, MCI, and diagnostics as keywords, the data were searched from PubMed, EMbase and Cochrane library database, and the results of different queries were combined using Boolean operator AND. Also, the reference lists of the included studies were manually searched to identify any relevant articles.

### Inclusion and exclusion criteria

Among the collected studies, the research complying with the following inclusion criteria was enrolled for meta-analysis:

- observational project: a meta-analysis of MRI-based deep learning diagnosi s of AD and MCI;
- subjects: patients with AD and MCI;
- intervention (subgroups): deep learning modeling group; and
- evaluation metrics: sensitivity, specificity, positive likelihood ratio, negative likelihood ratio, diagnostic odds ratio, and area under the receiver operating characteristic curve (AUROC).

Exclusion criteria included magazine publication types (e.g., reviews, letters to the editors, editorials, conference abstracts); and scientific publication types, such as case reports, meta-analyses, literature reviews, and cross-sectional studies; full-text not available; data not extracted; and participants with AD and other mental illnesses. Also, the subjects must not have been the study of MRI-based deep learning for differential diagnosis of AD and MCI.

### Data extraction

A standardized form was developed to capture information including first author, country, year of publication, type of AI model, number of patients, patient characteristics (mean/median age, gender), and diagnostic efficiency. The above information was extracted independently by two evaluators from each eligible study. In addition, data such as AUROC, sensitivity, specificity, and accuracy were extracted for data processing and forest mapping.

### Quality assessment

The risk of bias was initially assessed independently by two evaluators. Then, a third evaluator was responsible for reviewing each study using the Quality Assessment of Diagnostic Accuracy Studies-2 (QUADAS-2) guidelines. 16 The QUADAS-2 tool could be used to assign the risk of bias to “low”, “high”, or “uncertain” based on a “yes”, “no”, or “uncertain” response to the relevant marked question contained in each section. For example, if the answer to all landmark questions in the scope was “yes”, then it could be rated as a low risk of bias; if the answer to all information questions was “no”, then the risk of bias was rated as “high”. To ensure the accuracy and reliability of the information gathered, all data were initially extracted independently by two evaluators. In cases where discrepancies arose, a predefined protocol was employed to resolve these differences. This involved a detailed discussion between the evaluators to reach a consensus. If consensus could not be achieved, a third, senior evaluator was consulted to make the final decision. This rigorous procedure was designed to minimize subjective interpretation and bias, thereby enhancing the reliability of the data extraction process.

### Statistical analysis

In this study, Stata 16.0 software was used to calculate the combined sensitivity and specificity of each study to assess the accuracy of the meta-analysis, and the combined positive likelihood ratio, combined negative likelihood ratio, and combined diagnostic ratio were also calculated. The total receiver operating characteristic (ROC) curve was also drawn to calculate AUC. Hierarchical summary of receiver operating characteristic (HSROC) curves with credible and predictive regions were simultaneously constructed. The Spearman's correlation coefficient test was calculated to determine whether there was heterogeneity caused by the threshold effect. Deek's funnel plot was plotted to assess the publication bias more intuitively. *p* < 0.05 indicated a statistically significant difference.

## RESULTS

### Literature screening

**Figure 1:**
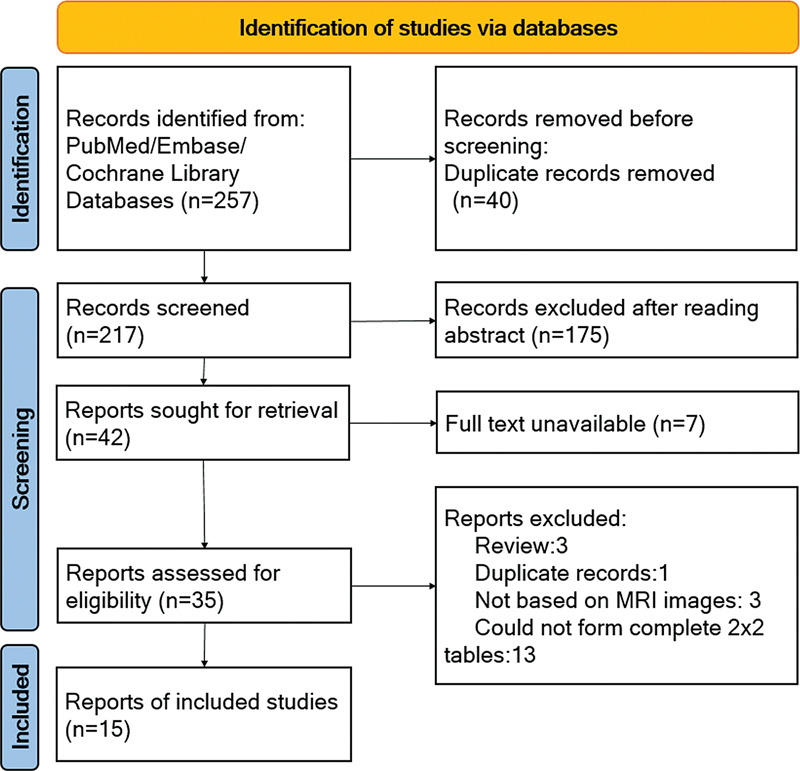
PRISMA flowchart outlines the process of studies' selection.

The literature search process is shown in the PRISMA flowchart in Figure 1 . There were 257 studies identified, and 40 duplicate studies were removed. The remaining studies were screened. Of them, 175 did not meet the inclusion criteria based on title and abstract. The remaining 42 complete manuscripts were individually assessed, and finally, 35 studies were eligible for inclusion in our systematic review. 15 papers were available for meta-analysis, 17 18 19 20 21 22 23 24 25 26 27 28 29 30 31 and 20 articles were excluded due to insufficient data.

**Figure 2:**
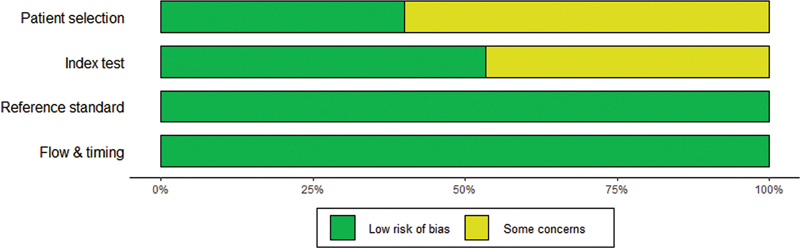
Results of quality evaluation of the included literature. The quality of the literature was assessed by the Quality Assessment of Diagnostic Accuracy Studies-2 (QUADAS-2). The color coding represents the assessed risk of bias, with green indicating a low risk of bias and yellow representing some concerns about potential bias.

The included studies were published from 2019 to 2023. Among them, 1 study involved 3 research projects, and 1 study included 2 research projects. Therefore, we collected data from 18 studies. Of them, 6 of these studies were conducted in China, 4 in South Korea, 2 each in Spain, Italy, and Iran, and 1 each in India and Singapore ( Table 1 ). The adopted AI learning models in these studies included CNN (EfficientNet), 3D CNN, CNN + Transfer Learning, Long Short-Term Memory (LSTM) networks + CNN, CNN + iterated Radio Frequency (RF), 2D CNNs, Support Vector Machine (SVM) + Auto-Encoder Neural Networks, CNN + XGBoost, 3D CNN + SVM and 3D CNN + Computer Aided Engineering (CAE) ( Table 2 ). The results of the quality assessment of the included literature are shown in Table 3 and Figure 2 .

**Table 1:** Basic characteristics of the included studies

Author	Year	Country	MRI	Sample size		Database	Type of research
				AD	MCI		
Li H 26	2023	China	structural Magnetic Resonance Imaging(sMRI)	151	142	ADNI	Retrospective
Agarwal D 17	2023	Spain	T1-weighted brain MRI	229	229	ADNI	Retrospective
Tanveer M 30	2022	India	T1-weighted brain MRI	187	398	ADNI	Retrospective
Gao L 23	2022	China	T1-weighted brain MRI	154	145	ANDI	Retrospective
Chen X 21	2022	China	T2-weighted brain MRI	43	97	ADNI	Retrospective
Agarwal D 18	2022	Spain	T1-weighted brain MRI	245	229	ADNI + IXI	Retrospective
Mehmood A 27	2021	China	T1-weighted brain MRI	85	70	ADNI	Retrospective
Kang W 25	2021	China	T1-weighted brain MRI	187	382	ADNI	Retrospective
Hedayati R 24	2021	Iran	Functional magnetic resonance imaging (fMRI)	100	100	ADNI	Retrospective
Akramifard H 19	2021	Iran	Not explicitly stated	156	338	ADNI	Retrospective
Suh C H 29	2020	South Korea	T1-weighted brain MRI	161	363	Asan Medical Center	Retrospective
Suh C H 29	2020	South Korea	T1-weighted brain MRI	68	63	Kyung Hee University Hospital at Gangdong	Retrospective
Suh C H 29	2020	South Korea	T1-weighted brain MRI	178	317	ADNI	Retrospective
Feng W 22	2020	China	T1-weighted brain MRI	130	133	ADNI	Retrospective
Wee C Y 31	2019	Singapore	T1-weighted brain MRI	592	899	ADNI	Retrospective
Oh K 28	2019	South Korea	T1-weighted brain MRI	198	101	ADNI	Retrospective
Basaia S 20	2019	Italy	T1 and T2-weighted brain MRI	418	533	ADNI + Milan dataset	Retrospective
Basaia S 20	2019	Italy	T1 and T2-weighted MRI	294	510	ADNI	Retrospective

Abbreviations: AD, Alzheimer's disease; ADNI, Alzheimer's Disease Neuroimaging Initiative; MCI, mild cognitive impairment; MRI, magnetic resonance imaging.

**Table 2:** Characteristics of artificial intelligence learning models of the included literature

Author	Year	Modeling	Test Set	TP	FP	FN	TN
Li H 26	2023	CNN (EfficientNet)	AD:30,MCI: 30	27	2	3	28
Agarwal D 17	2023	3D CNN (EfficientNet-B0)	AD:29,MCI: 29	29	4	0	25
Tanveer M 30	2022	CNN + Transfer Learning	20%	37	1	1	79
Gao L 23	2022	LSTM networks + CNN	AD:31,MCI:18	25	2	6	16
Chen X 21	2022	CNN + iterated RF	AD:30,MCI: 30	28	2	2	28
Agarwal D 18	2022	3D CNN (DenseNet264)	AD:29,MCI: 29	27	10	2	19
Mehmood A 27	2021	CNN + Transfer Learning	20%	13	2	2	12
Kang W 25	2021	2D CNNs	20%	26	35	12	42
Hedayati R 24	2021	CNN	AD:20,MCI: 20	17	1	3	19
Akramifard H 19	2021	SVM + Auto-Encoder Neural Networks	10%	8	6	8	28
Suh C H 29	2020	CNN + XGBoost	20%	23	19	10	54
Suh C H 29	2020	CNN + XGBoost	20%	10	3	4	10
Suh C H 29	2020	CNN + XGBoost	20%	24	19	12	45
Feng W 22	2020	3D CNN + SVM	AD:23,MCI: 24	22	1	1	23
Wee C Y 31	2019	CNN	10%	42	13	18	77
Oh K 28	2019	3D CNN + CAE	10%	15	3	5	8
Basaia S 20	2019	3D CNN	10%	35	6	7	48
Basaia S 20	2019	3D CNN	10%	25	6	5	45

Abbreviations: CAE, Computer Aided Engineering; CNN, Convolutional Neural Networks; LSTM, Long Short-Term Memory; RF, Radio Frequency; SVM, Support Vector Machine.

**Table 3:** Quality assessment results of the quality assessment of diagnostic accuracy studies 2 of the included literature

Author	Year	Patient selection	Index test	Reference standard	Flow and timing
Li H 26	2023	low	unclear	low	low
Agarwal D 17	2023	unclear	unclear	low	low
Tanveer M 30	2022	unclear	low	low	low
Gao L 23	2022	unclear	unclear	low	low
Chen X 21	2022	unclear	unclear	low	low
Agarwal D 18	2022	unclear	unclear	low	low
Mehmood A 27	2021	low	low	low	low
Kang W 25	2021	unclear	low	low	low
Hedayati R 24	2021	unclear	unclear	low	low
Akramifard H 19	2021	low	low	low	low
Suh C H 29	2020	low	low	low	low
Feng W 22	2020	unclear	unclear	low	low
Wee C Y 31	2019	low	low	low	low
Oh K 28	2019	unclear	low	low	low
Basaia S 20	2019	low	low	low	low

### Meta-analysis results

**Figure 3:**
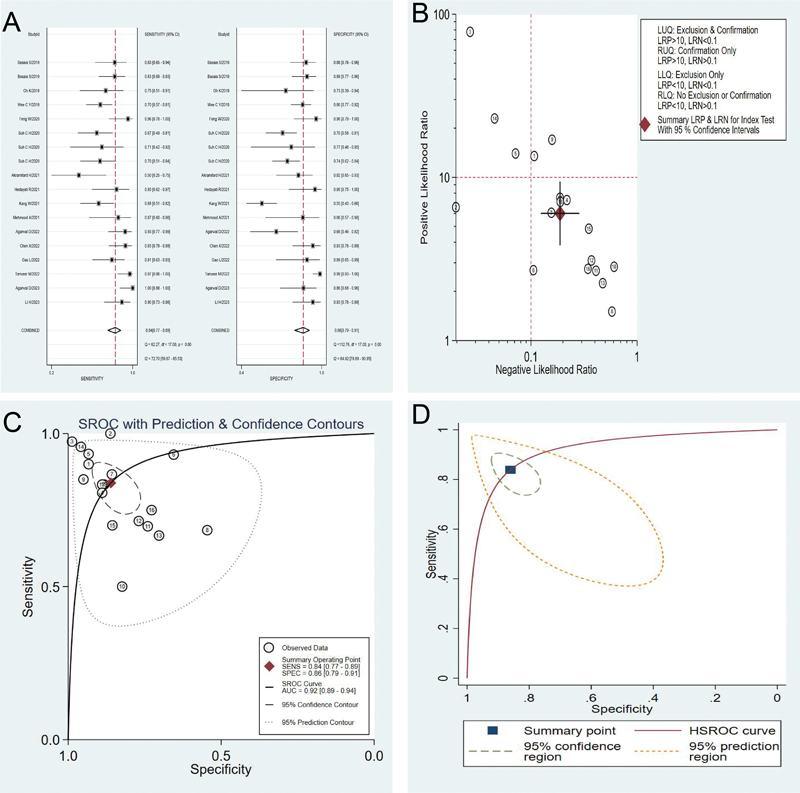
Meta-analysis results. ( **A** ) Combined sensitivity and specificity forest maps for MRI-based deep learning to diagnose Alzheimer's disease and mild cognitive impairment; ( **B** ) Combined positive likelihood ratio and negative likelihood ratio for diagnosis of Alzheimer's disease and mild cognitive impairment using MRI-based deep learning; ( **C** ) The Receiver Operating Characteristic Curve of MRI-based deep learning for diagnosing Alzheimer's disease and mild cognitive impairment; ( **D** ) Hierarchical summary receiver operating characteristic curves for MRI-based deep learning to diagnose Alzheimer's disease and mild cognitive impairment

Among the 18 studies included in this research, the Spearman correlation coefficient test showed that the coefficient was -0.6506, ( *p* = 0.0035), suggesting the presence of a threshold effect. A random-effects model was adopted for meta-analysis owing to the heterogeneity of the included studies (I ^2^ = 31%, 95% CI = 0–100). The sensitivity and specificity of deep learning in MRI for diagnosing AD and MCI were combined and analyzed. The results suggested that the combined sensitivity, combined specificity, positive likelihood ratio, negative likelihood ratio, and diagnostic odds ratio were 0.84 (95% CI: 0.77–0.89), 0.86 (95% CI: 0.79–0.91), and 6.0 (95% CI: 3.8–9.4), 0.19 (95% CI: 0.12–0.28), 32 (95% CI: 14–72), respectively ( Figures 3A and B ). In addition, the AUROC curve ( Figure 3C ) was 0.92 (95% CI: 0.89–0.94), indicating that MRI-based deep learning had high accuracy in differentiating the diagnosis for AD and MCI in terms of sensitivity and specificity. Then, HSROC analysis was performed to avoid the threshold effect that could adversely affect the results ( Figure 3D ). The Q-point of the HSROC curve was 3.463. The results of HSROC suggested that MRI-based deep learning exhibited good sensitivity and specificity in diagnosing AD and MCI, as well as a high diagnostic odds ratio.

### Analysis of publication bias in the literature

**Figure 4:**
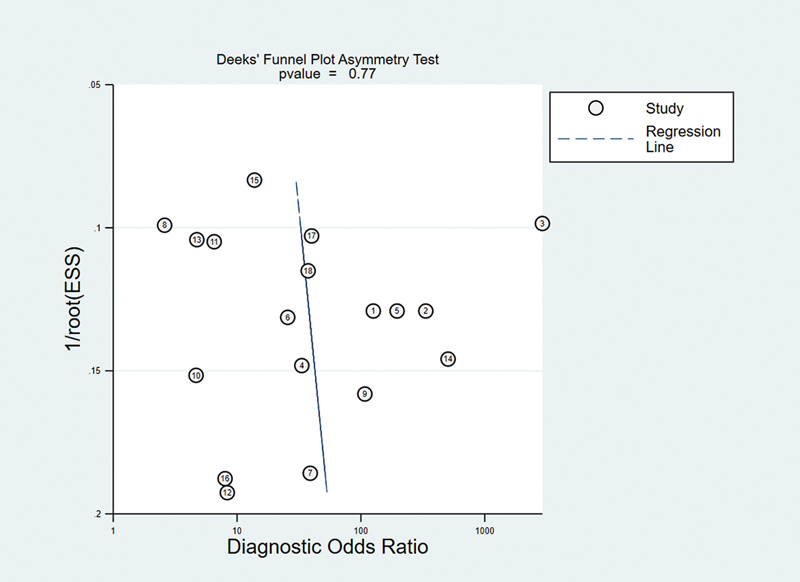
Deek's funnel plot for diagnosis of Alzheimer's disease and mild cognitive impairment based on MRI deep learning. This plot is a tool used to visually inspect for the presence of publication bias in meta-analyses. Each point represents a study included in the meta-analysis, plotted according to its effect size against a measure of precision (the inverse of the standard error). The funnel plot's asymmetry is tested using Deek's test, where a symmetrical distribution suggests the absence of publication bias, and asymmetry may indicate its presence.

Deek's funnel plot was adopted to assess whether there was publication bias in the collected literature. The results showed ( Figure 4 **)** that the collected studies were approximately distributed along the central axis of symmetry in Deek's funnel plot ( *p* = 0.77). This data illustrated the absence of publication bias in our collected literature.

### Subgroup analysis

As shown in Table 1 , structural MRI (sMRI) was used in 1 study, T1-weighted brain MR images 17 18 22 23 25 27 28 29 30 31 were used in 12 studies, and T1 and T2-weighted brain MR images were used in 2 studies. 20 In addition, there was 1 study, using T2-weighted brain MR images 21 and fMRI 24 for diagnosis, and 1 study did not specify the diagnostic method. 19 These diagnostic methods could be reduced to two MRI techniques, namely, the T1-weighted MRI alone group (containing 12 studies) and the non-T1-weighted MRI alone group (containing 6 studies).

According to Table 4 , the combined sensitivity of T1-weighted MRI was 0.84 (95% CI: 0.74–0.91), which was similar to that of non-T1-weighted MRI (0.84, 95% CI: 0.72–0.91), but there was no significant difference. Additionally, the combined specificity, negative likelihood ratio, positive likelihood ratio, and combined diagnostic ratio of T1-weighted MRI and non-T1-weighted MRI were not significantly different. However, the positive likelihood ratio of T1-weighted MRI was 5, while the positive likelihood ratio of non-T1-weighted MRI was 8.3, indicating that the positive likelihood of non-T1-weighted MRI was higher. Therefore, when the result was positive, the patients may be diagnosed as positive more accurately by non-T1-weighted MR. Moreover, the AUC for T1-weighted MRI was 0.91, and for non-T1-weighted MRI was 0.93, both indicating good diagnostic accuracy. These data suggested that the two MRI techniques were similar in terms of sensitivity, negative likelihood ratio, and AUC, but non-T1-weighted MRI had a slight advantage in specificity and positive likelihood ratio. Nevertheless, these differences may be not statistically significant due to the overlapping of CI.

**Table 4:** Subgroup analysis

	T1-weighted MRI alone	Non-T1-weighted MRI alone
Number of studies	12	6
Combined sensitivity (95% CI)	0.84 (0.74, 0.91)	0.84 (0.72, 0.91)
Combined specificity (95% CI)	0.83 (0.73, 0.90)	0.9 (0.84, 0.94)
Positive likelihood ratio (95% CI)	5 (2.8, 8.9)	8.3 (5.0, 14.0)
Negative likelihood ratio (95% CI)	0.19 (0.10, 0.34)	0.18 (0.10, 0.33)
The combined diagnostic odds ratio (95% CI)	26 (9, 79)	46 (17, 127)
AUC	0.91 (0.88, 0.93)	0.93 (0.91, 0.95)

Abbreviations: AUC, Area Under Curve; CI, confidence interval; MRI, magnetic resonance imaging.

## DISCUSSION

The data of many images, such as MRI, computed tomography, and positron emission tomography, need to be collected and generated during the diagnosis and treatment of AD or MCI. 32 Clinically, medical staff usually evaluate this data subjectively and formulate treatment plans based on experience. Accurate early diagnosis of AD and MCI is essential for treatment. However, the features of the imaging data observed only relying on the naked eye of the medical staff were limited and may make many potential imaging data not fully revealed. 33 In recent years, many researchers have attempted to use sophisticated mathematical and statistical algorithms to extract hard-to-observe quantitative information to increase the diagnostic accuracy of AD and the potential to predict the worsening progression of MCI. 34

In this study, we collected 15 articles from 2019 to 2023 and analyzed the diagnostic accuracy of deep learning methods based on brain MRI data in AD and MCI through meta-analysis. These studies were performed based on convolutional neural network (CNN) in conjunction with different algorithms. CNN involves a great many deep learning techniques and has been proven to be effective in diagnosing non-dementia, particularly mild dementia, mild dementia, and moderate dementia. 35 Jo T et al. collected the deep learning papers on AD published from 2013 to 2018 and performed a meta-analysis. In their study, they concluded that deep learning had 83.7% accuracy for AD classification; the accuracy for predicting progression from MCI to AD was as high as 96.0%. 36 Spasov et al. collected the data of 192 patients with AD and 409 patients with MCI and then built a deep learning algorithm to distinguish the MCI patients developing into AD within 3 years from those MCI patients with stable condition i n the same period; the AUC was 0.925, the 10-fold cross-validated accuracy was 86%, the sensitivity was 87.5%, and the specificity of 85%. 37 Wei et al. utilized three-dimensional convolutional neural networks (3D-CNNs) and MRI to build binary and ternary disease classification models. Through these models, they observed that the ternary classification accuracy of the 3D-CNN-support vector machine (3D-CNN-SVM) for the diagnosis of MCI and AD were 96.82% and 96.73%, respectively. 22 The above findings revealed the significance of deep learning in improving the accuracy of MRI-based diagnosis of AD and MCI. The meta-analysis of this study showed that the deep learning of MRI had good sensitivity and specificity in diagnosing AD and MCI overall.

The variability across different deep learning architectures presents both challenges and opportunities for enhancing diagnostic performance in neurodegenerative diseases. Studies have demonstrated that the choice of architecture, from CNNs to more complex models like Recurrent Neural Networks (RNNs) and their hybrids, can significantly affect the model's ability to learn and generalize from neuroimaging data. 38 Furthermore, the diversity in training datasets—spanning various demographics, disease stages, and imaging protocols—introduces additional layers of variability that can influence diagnostic outcomes. 39 Notwithstanding these challenges, adopting strategies such as transfer learning, data augmentation, and ensemble learning models offers promising pathways to achieving more consistent and reliable diagnostic predictions across diverse clinical settings. 40

In the literature we collected, the uncertainty of heterogeneity was large, but there was no publication bias. Moreover, we did not find any significant difference in the diagnosis of AD and MCI between the T1-weighted MRI alone and non-T1-weighted MRI alone. However, it cannot be ignored that multiple learning models were used in the literature collected in this paper. These learning models inevitably induced data bias and then affected the comparison of the overall diagnostic performance. The literature collected in this study was also retrospective, and most of the studies had no directly available data and deep learning codes. Moreover, only internal validation or resampling methods were used to judge the accuracy of deep learning. Such validation methods lack generalization, and the internal validation tends to overestimate the AUC, especially for out-of-distribution detection on 3D medical images. 41 Simultaneously, what could not be ignored was that the insufficiency of prospective studies on deep learning for AD and MCI limited the integration of AI models with the clinical setting. 42 Therefore, externally validated predictive models using images from different hospitals are required to create reliable estimates of the performance level at other sites. In addition, reports with incomplete data were removed during the literature screening, which might affect the estimates of diagnostic performance. 43 Besides, the results of the calculations may be geographically biased, since the included studies were from geographically diverse quantitative distributions; moreover, the type of scanner used for diagnosis, the imaging protocol, and the diagnostic criteria for AD and MCI may also affect the accuracy of results. 44 However, deep learning itself has great potential because it can continuously improve the algorithms to increase the accuracy. Therefore, future research should prioritize the development of standardized protocols for data acquisition and preprocessing. Additionally, there's a pressing need for collaborative efforts to establish large, annotated datasets that reflect the diversity of the global population. Moreover, the exploration of federated learning approaches, where AI models are collaboratively trained across multiple institutions while keeping data localized, offers a promising direction.

In summary, our meta-analysis underscores the significant potential of deep learning algorithms applied to MRI images in enhancing the diagnostic accuracy for AD and MCI. The analysis revealed that deep learning models exhibit high sensitivity and specificity, indicating their reliability in identifying AD and MCI from neuroimaging data. These findings highlight the transformative impact of AI in the field of neurology, offering a promising tool for early and accurate disease diagnosis. Moving forward, it is imperative for future research to focus on addressing the challenges of model variability and data heterogeneity to further refine AI applications in medical diagnostics. Clinically, integrating AI into diagnostic workflows has the potential to revolutionize patient care by enabling timely and personalized treatment interventions. This research not only contributes to the existing body of knowledge but also lays a foundational path for leveraging AI to improve outcomes for individuals with neurodegenerative disorders.

In conclusion, deep learning models based on MRI images have the potential to improve diagnostic accuracy in AD and MCI, which can not only provide clinicians with individualized preoperative noninvasive auxiliary prediction tools but also increase the early diagnosis rate of the patients with AD and MCI to develop better treatment strategies. Furthermore, with the continuous improvement of deep algorithms, more effective algorithms may be developed to further improve the diagnostic accuracy of AI in AD and MCI.
